# Championing Older Adults in Climate Empowerment (COALESCE) Phase 1 - A Participatory Rapid Realist Review

**DOI:** 10.1093/geroni/igaf122.3727

**Published:** 2025-12-31

**Authors:** Mei Fang, Rebecca White, Farnoush Mansourian, Sreya Ajay, Sharon Sa, Dannah DeSouza, Claire Dreyfuss, Elise Stone

**Affiliations:** Simon Fraser University, Vancouver, British Columbia, Canada; Simon Fraser University, Vancouver, British Columbia, Canada; Simon Fraser University, Vancouver, British Columbia, Canada; SFU, Vancouver, British Columbia, Canada; Simon Fraser University, Vancouver, British Columbia, Canada; Simon Fraser University, Vancouver, British Columbia, Canada; Simon Fraser University, Vancouver, British Columbia, Canada; Simon Fraser University, Vancouver, British Columbia, Canada

## Abstract

**Background:**

The COALESCE Project (Phase I) investigates how older adults experience and prioritise the effects of climate breakdown. By engaging them as citizen science co-researchers, the project aims to co-develop resilience strategies tailored to their needs. Partnering older adults with graduate students fostered intergenerational exchange, enhancing research capacity and generating practical interventions to address severe weather impacts on ageing populations.

**Methods:**

A participant-led rapid realist review (PRRR) was piloted to explore how best to identify climate change priorities and effective interventions. Five graduate students and seven older adult co-researchers were trained to collaboratively undertake a time-limited PRRR, including literature search, data extraction, and synthesis. Student–older adult pairs conducted targeted searches of academic and grey literature on climate-related impacts and resilience strategies for older people. Guided by older adults’ lived experiences, the review process integrated life-course interviews and deliberative dialogues, enabling reflection, critique, and adaptation of the PRRR method.

**Findings:**

The pilot showed that effective co-research requires working to the unique strengths of both older adults and students. Rather than duplicating tasks, collaboration was optimised when students applied academic research skills alongside older adults’ lived experience and contextual knowledge, ensuring shared responsibility and ownership of the review.

**Discussion:**

This intergenerational co-research model deepened understanding of climate impacts on older people, built mutual research capacity, and empowered participants. Critically, the project demonstrated the potential of participatory realist reviews to co-produce knowledge and inform interventions, ensuring older adults define their own climate change contexts and resilience needs.

